# Effects of axial length on retinal nerve fiber layer and macular ganglion cell-inner plexiform layer measured by spectral-domain OCT

**DOI:** 10.5935/0004-2749.20200039

**Published:** 2020

**Authors:** Carolina Lampert Monte Francisconi, Mário Bernardes Wagner, Roberto Vanin Pinto Ribeiro, André Moraes Freitas

**Affiliations:** 1 Department of Ophthalmology, Universidade Federal de Ciências da Saúde de Porto Alegre, Porto Alegre, RS, Brazil; 2 Department of Ophthalmology and Visual Sciences, Dalhousie University, Halifax, NS, Canada; 3 Biostatistics and Epidemiology Department, Universidade Federal do Rio Grande do Sul, Porto Alegre, RS, Brazil; 4 Institute of Medical Science, University of Toronto, Toronto, Canadá

**Keywords:** Tomography, optical coherence, Retinal ganglion cells, Axial length, eye, Tomografia de coerência óptica, Células ganglionares da retina, Comprimento axial do olho

## Abstract

**Purpose:**

To evaluate the influence of ocular axial length on circumpapillary retinal nerve fiber layer and ganglion cell-inner plexiform layer thickness in healthy eyes after correcting for ocular magnification effect.

**Methods:**

In this cross-sectional study, we evaluated 120 eyes from 60 volunteer participants (myopes, emmetropes, and hyperopes). The thickness of the circumpapillary retinal nerve fiber layer and ganglion cell-inner plexiform layer were measured using the spectral optical coherence tomography (OCT)-Cirrus HD-OCT and correlated with ocular axial length. Adjustment for ocular magnification was performed by applying Littmann’s formula.

**Results:**

Before the adjustment for ocular magnification, age-adjusted mixed models analysis demonstrated a significant negative correlation between axial length and average circumpapillary retinal nerve fiber layer thickness (r=-0.43, p<0.001), inferior circumpapillary retinal nerve fiber layer thickness (r=-0.46, p<0.001), superior circumpapillary retinal nerve fiber layer thickness (r=-0.31, p<0.05), nasal circumpapillary retinal nerve fiber layer thickness (r=-0.35, p<0.001), and average ganglion cell-inner plexiform layer thickness (r=-0.35, p<0.05). However, after correcting for magnification effect, the results were considerably different, revealing only a positive correlation between axial length and temporal retinal nerve fiber layer thickness (r=0.42, p<0.001). Additionally, we demonstrated a positive correlation between axial length and average ganglion cell-inner plexiform layer thickness (r=0.48, p<0.001). All other correlations were not found to be statistically significant.

**Conclusions:**

Before adjustment for ocular magnification, axial length was negatively correlated with circumpapillary retinal nerve fiber layer and ganglion cell-inner plexiform layer thickness measured by Cirrus-OCT. We attributed this effect to ocular magnification associated with greater axial lengths, which was corrected with the Littman’s formula. Further studies are required to investigate the impact of ocular magnification correction on the diagnostic accuracy of Cirrus-OCT.

## Abstract (pt)

**Objetivo:**

Avaliar a influência do comprimento axial ocular na espessura da camada de fibras nervosas da retina peripapilar e na espessura da camada de células ganglionares-plexiforme interna em olhos saudáveis após correção para efeito de magnificação ocular.

**Métodos:**

Neste estudo transversal, avaliamos 120 olhos de 60 participantes voluntários (míopes, emétropes e hipermétropes). A espessura da camada de fibras nervosas da retina peripapilar e da camada de células ganglionares-plexiforme interna foram medidas usando a tomografia de coerência óptica espectral (OCT)-Cirrus HD-OCT e correlacionada com o comprimento axial ocular. O ajuste para a magnificação ocular foi realizado aplicando a fórmula de Littmann.

**Resultados:**

Antes do ajuste para magnificação ocular, a análise de modelos mistos ajustada por idade demonstrou uma correlação negativa significante entre o comprimento axial e a espessura média da camada de fibras nervosas da retina peripapilar (r=-0,43; p<0,001), espessura da camada de fibras nervosas da retina peripapilar inferior (r=-0,46; p <0,001), espessura da camada de fibras nervosas da retina peripapilar superior (r=-0,31; p<0,05), espessura da camada de fibras nervosas da retina peripapilar nasal (r=-0,35; p<0,001) e espessura média das células ganglionares-plexiforme interna (r=-0,35; p<0,05). No entanto, após a correção do efeito de magnificação, os resultados foram consideravelmente diferentes, revelando apenas uma correlação positiva entre o comprimento axial e a espessura temporal da camada de fibras nervosas da retina (r=0,42; p<0,001). Além disso, demonstramos uma correlação positiva entre o comprimento axial e a espessura média das células ganglionares-plexiforme interna (r=0,48; p<0,001). Todas as outras correlações não foram consideradas estatisticamente significativas.

**Conclusão:**

Antes do ajuste para o efeito de magnificação ocular, o comprimento axial estava ne gativamente correlacionado com a espessura da camada de fibras nervosas da retina peripapilar e das células ganglionares-plexiforme interna medido pelo Cirrus-OCT. Atribuimos esse efeito à magnificação ocular associada a comprimentos axiais maiores, o que foi corrigido com a fórmula de Littman. Mais estudos são necessários para investigar o impacto da correção da magnificação ocular na acurácia diagnóstica do Cirrus-OCT.

## INTRODUCTION

Progressive thinning of the circumpapillary retinal nerve fiber layer (cpRNFL) due to ganglion cell death is a characteristic feature of glaucoma, which has been harnessed for aiding diagnosis and monitoring of glaucoma^(1,2)^. Loss of cpRNFL is thought to precede optic nerve head (ONH) and visual field (VF) abnormalities since this progressive thinning was observed in up to 60% of eyes approximately six years prior to any detectable VF defects^(2)^. Ocular coherence tomography (OCT) has been demonstrated to be ideal for the detection of cpRNFL damage and thereby aiding in the diagnosis of early-stage glaucoma.

Recently, macular ganglion cell analysis (GCA) was found to be helpful for the early detection of glaucoma. Cirrus HD (high-definition) OCT enables a GCA for the determination of ganglion cell-inner plexiform layer (GCIPL) thickness. Assessment of macular GCIPL thickness and GCA maps have proven to be ideal tests for the early detection of glaucoma^(3,4)^, even in highly myopic pa tients^(5)^. However, abnormalities have also been observed in non-glau co matous eyes, especially in those with high myopia^(6,7)^. Myopia is a risk factor for glaucoma, and myopic fundus changes often complicates the diagnosis and management of glaucoma^(8-10)^. Despite OCT being a modern imaging device that measures thickness of cpRNFL and GCIPL, the Cirrus-OCT software is packaged with cpRNFL measurements from a normal population database^(11)^, and therefore does not distinguish individuals with moderate or high degrees of myopia, which may lead to an inaccurate diagnosis. In this regard, proper interpretation of the data obtained by Cirrus HD-OCT examination requires an evaluation of the influence of ocular axial length on cpRNFL and GCIPL thickness, which despite an extensive investigation, presents conflicting data in the literature^(7,12-14)^. These disagreements could be attributed to differences in study methodology, such as the OCT instrument used, the adjustment for age or ocular magnification effect, the population evaluated and the method of analysis. Therefore, the aim of the present study was to evaluate the influence of different ocular axial lengths on thickness of cpRNFL and GCIPL and their correlations in healthy eyes, in healthy eyes, considering correction for ocular magnification effect.

## METHODS

The study protocol and informed consent were appro ved by the institutional Ethics and Research Committee of Irmandade Santa Casa de Misericórdia de Porto Alegre Hospital. The study adhered to the tenets of the Declaration of Helsinki.

Between June 2013 and August 2015, myopic, emmetropic and hyperopic volunteers over the age of 18 and without other ocular pathologies were screened for participation in this cross-sectional study. We excluded individuals with diabetes, cataracts, previous ocular surgery, inflammation or trauma, neurological diseases, eye conditions that may affect the cpRNFL or VF, visual acuity with best correction worse than 20/30, glaucoma, intraocular pressure greater than 21 mmHg, contraindications to mydriatic eye drops, VF abnormalities, and current or previous prolonged use of corticosteroids. A VF was considered abnormal if the Glaucoma Hemifield Test results were outside of normal limits and/or the corrected pattern standard deviation was p<0.05%, as confirmed by a reliable examination (false-positive/negatives <15%, fixation losses <15%)^(15)^.

All subjects underwent a thorough ophthalmic examination to confirm the absence of ocular pathologies except for refractive error, and included the following assessments: visual acuity by Snellen method and refractive error with a model Topcon Model KR 8900 autorefractor keratometer (refractometry was converted to spherical equivalents); Perkins applanation tonometry; anterior segment examination by slit-lamp biomicroscopy; ONH evaluation (including cup-to-disk ratios and presence of disk tilt and/or peripapillary atrophy) and fundus examination with a 90-diopter lens; 30.2 Swedish Interactive Threshold Algorithm standard automated VF test using a Humphrey Visual Field Analyzer (Carl Zeiss Meditec), axial length biometry (LENSTAR LS900^®^, Haag-Streit); and circumpapillary RNFL and macular measurement using Cirrus 4000 HD-OCT (Carl Zeiss Meditec, Dublin, CA).

Patients who met the study criteria underwent pharmacological dilation of the pupil and then eyes were scanned with Cirrus HD-OCT system. A 200 × 200 cube optic disc scan and 512 × 128 macular cube scan were obtained. Images with signal strength (an indicator of image quality) <7 were discarded and a new image was obtained until signal strength of at least seven was achieved. The average cpRNFL thickness in each quadrant (superior, temporal, nasal, and inferior) was recorded. No participant showed peripapillary atrophy crossing the scanning circle. All scans were obtained by a single operator with more than one year experience with Cirrus-OCT operation.

The built-in algorithms of Cirrus HD-OCT (version 6.5.0.772) are capable of automatically identifying the vitreoretinal interface and posterior boundary of the cpRNFL and can subsequently calculate its thickness. The GCA algorithm identifies the outer boundary of the macular RNFL and the outer boundary of the inner plexiform layer (IPL). The difference between the RNFL and the IPL outer boundary segmentation yields the GCIPL thickness^(6)^. The average, minimum, and sectoral (superotemporal, superior, superonasal, inferonasal, inferior, and inferotemporal) GCIPL thicknesses are measured in an elliptical annulus with a vertical outer radius of 2.0 mm and horizontal radius of 2.4 mm.

Adjustment for ocular magnification followed the protocol described by Bennet et al.^(16)^. In brief, the relationship between the measurements obtained by an imaging system and the actual fundus dimension can be expressed as *t= p* ⋅ *q* ⋅ *s*, where *t* is the actual fundus dimension, *s* is the measurement obtained using OCT, *p* is the magnification factor for the camera of the imaging system, and *q* is the magnification factor related to the eye^(16,17)^. The OCT system has a camera magnification factor (*p*) of 3.382^(17,18)^ and the formula for obtaining the eye magnification factor (*q*) is *q*= *0.01306 (axial length -1.82)*^(16,19)^.

### Statistical analyses

In order to detect correlation coefficients with a magnitude r ≥0.40 with 90% power and a type I error of 0.05, we calculated a sample size estimated at 60 observational units. Considering a design effect of 2.0 for clustered data we included 120 eyes (from 60 subjects). Normally distributed data were described using mean ± standard deviation. Categorical data were expressed using counts and percentages.

To account for the correlated observations (clustered data) we used random effects models to obtain correlation coefficients between measurements. Since the correlation coefficient is the slope (b) of the regression line when both the X and Y variables have been converted to z-scores, all quantitative measures were standardized prior to analysis. Significance level was set at α = 0.05. Analyses were conducted with SPSS version 22.0.

## RESULTS

Sixty-one subjects were enrolled in the study and one subject was excluded due to diagnosis of angioid streaks during fundus examination. A total of 120 eyes of 60 participants were included in the analyses. The population consisted mostly of women (67%), Caucasian ethnicity (98.4%), with a mean age of 28 ± 8 years (range 20-57). The average axial length was 23.96 ± 1.12 mm (range 21.39 to 26.72) and 44% had axial length of >24 mm. Mean spherical equivalent was -1.00 (range -5.25 to +5.75). None of the subjects had a peripapillary chorioretinal atrophy crossing the scanning circle.

**Figure 1:**
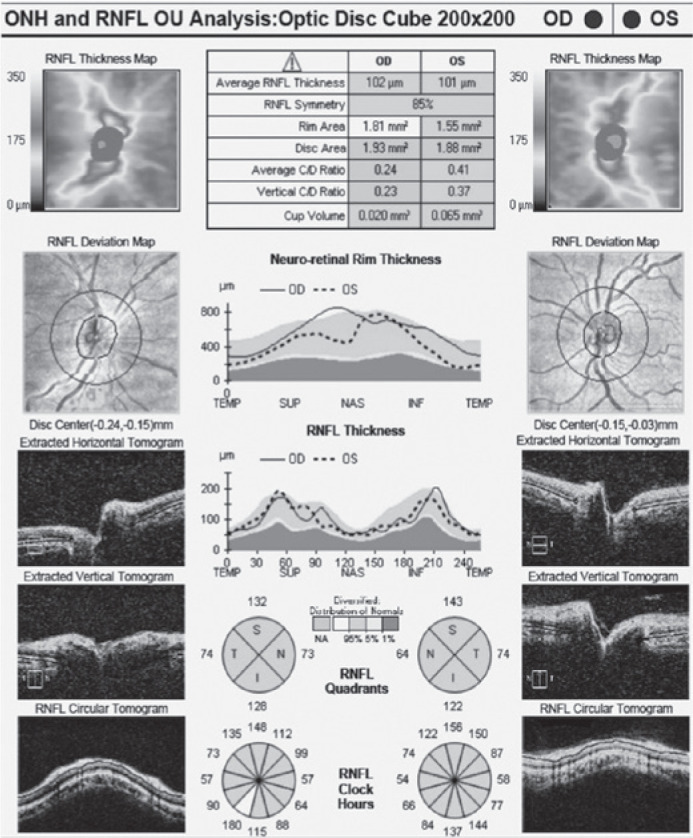
An example of circumpapillary retinal nerve fiber layer measurement in an emmetropic subject using Cirrus high-definition optical coherence tomography (Carl Zeiss Meditec).

**Figure 2:**
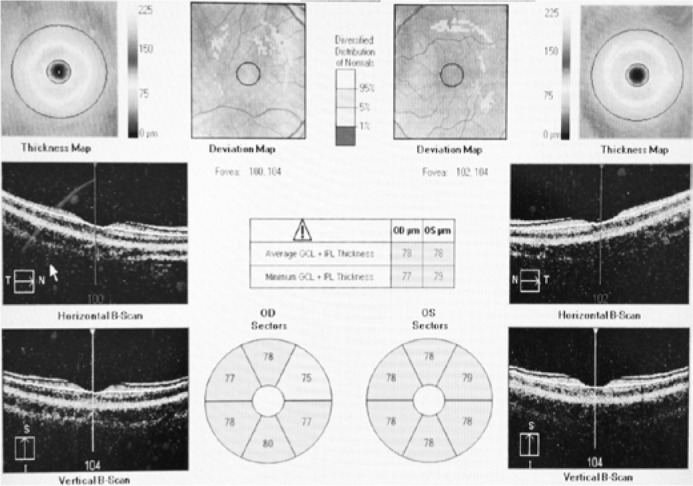
An example of ganglion cell analysis and ganglion cell-inner plexiform layer measurement in a low myopic subject using Cirrus high-definition optical coherence tomography (Carl Zeiss Meditec).

**Figure 3:**
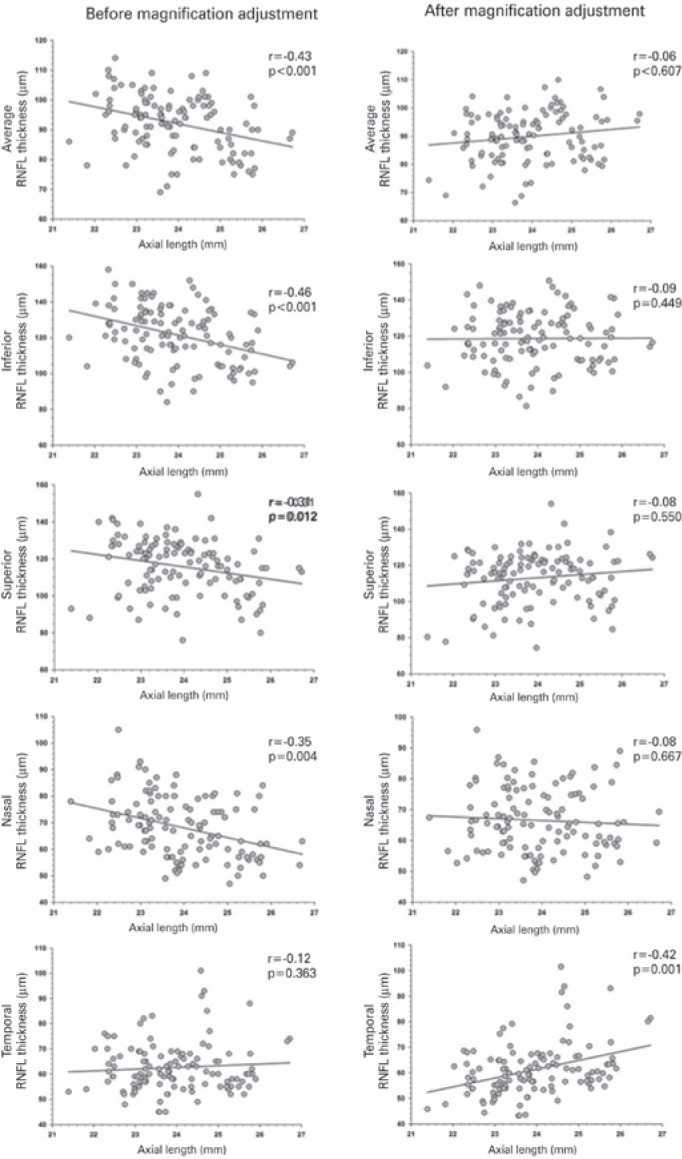
Scatterplot showing the relationship between retinal nerve fiber layer (RNFL) thickness and axial length before (left column) and after (right column) magnification adjustment. Note that RNFL becomes thinner with increased axial length; however, this effect disappears after the adjustment. A different pattern is observed with the temporal RNFL, which demonstrates a significant positive correlation after the adjustment.

Examples of measurements of cpRNFL (Figure 1) and GCIPL (Figure 2) by Cirrus-OCT are depicted. Before adjustment for ocular magnification (Figure 3) the mean RNFL thickness was 92 µm (range 69-114) and mean GCIPL thickness was 82 µm (range 69-94). Except for temporal cpRNFL (r=0.18, p=0.363), age-adjusted mixed models analysis demonstrated a significant negative correlation between axial length and average cpRNFL thickness (r=-0.43, p<0.001), inferior cpRNFL thickness (r=-0.46, p<0.001), superior cpRNFL thickness (r=-0.31, p<0.05), nasal cpRNFL thickness (r=-0.35, p<0.001), GCIPL thickness (r=-0.48, p<0.001), and average GCIPL thickness (r=-0.35, p<0.05). Individuals with axial length >24 mm showed a strong correlations between axial length and average cpRNFL (r=-0.45, p<0.05) and also between axial length axial length and GCIPL thickness (r=-0.48, p<0.001).

**Figure 4:**
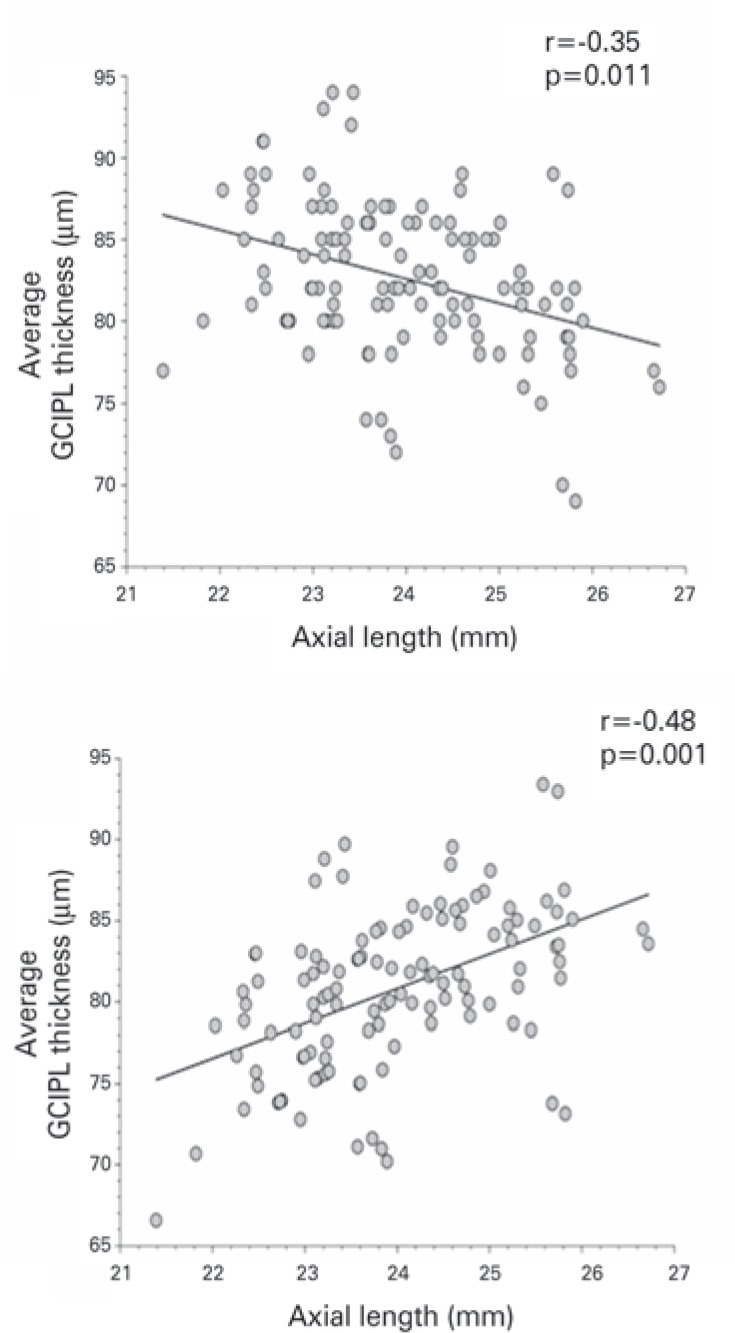
Scatterplot showing the relationship betw een ganglion cell-inner plexiform layer (GCIPL) thickness and axial length before (top image) and after (bottom image) magnification adjustment. Note that GCIPL becomes thinner with increased axial length; however, this effect reversed after the adjustment.

However, after the correction for magnification effect formula was applied (Figure 3 and 4), the results were considerably different. The mean RNFL thickness reduced to 90 µm (range 66-110) whereas the mean GCIPL thickness reduced to 81µm (range 67-93). Age-adjusted mixed model analysis revealed positive cor relations between axial length and temporal RNFL thickness (r=0.42, p<0.001) and average GCIPL thickness (r=0.48, p<0.001). The remaining correlations were not found to be statistically significant (Table 1). The mean values of cpRNFL and GCIPL thickness compared before and after magnification adjustment showed that all means were significantly different (Table 2).

**Table 1:** Correlation of axial length with thicknesses before and after magnification adjustments

	Before magnification adjustment		After magnification adjustment	
	CE	p value	CE	p value
RNFL thickness (µm)				
Average	**-0.43**	**<0.001**	**0.06**	**0.607**
Inferior quadrant	**-0.46**	**<0.001**	**-0.09**	**0.449**
Superior quadrant	**-0.31**	**0.012**	**0.08**	**0.550**
Nasal quadrant	**-0.35**	**0.004**	**-0.05**	**0.667**
Temporal quadrant	**0.12**	**0.363**	**0.42**	**0.001**
GCIPL thickness (pm)				
Average	**-0.35**	**0.011**	**0.48**	**<0.001**

CE= correlation of coefficient; GCIPL= ganglion cell-inner plexiform layer; RNFL= retinal nerve fiber layer. n=120.

**Table 2:** Comparison before and after magnification adjustments (mean ± SD)

	Before magnification adjustment	After magnification adjustment	p value
RNFL thickness (um) Average	92.18 ± 9.41	89.86 ± 8.73	<0.001
Inferior quadrant	121.80 ± 16.10	118.65 ± 14.56	<0.001
Superior quadrant	115.88 ± 14.90	112.97 ± 14.14	<0.001
Nasal quadrant	68.31 ± 11.42	66.50 ± 10.40	<0.001
Temporal quadrant	62.55 ± 9.90	61.11 ± 10.44	<0.001
GC1PL thickness (um) Average	82.62 ± 4.70	80.77 ± 4.90	<0.001

RNFL= retinal nerve fiber layer; SD= standard deviation; GCIPL= ganglion cell-inner plexiform layer. n=120.

## DISCUSSION

In the present study, the impact of axial length magnification effect Cirrus HD-OCT measurements of the cpRNFL and GCIPL was evaluated in a normal population, where we showed that, before correction for axial length magnification effect, the average, superior, inferior, and nasal cpRNFL were negatively correlated with axial length. The correlation was stronger at the inferior pole of the optic disc, but after correction only the temporal cpRNFL was positively correlated with axial length. The importance of the effect of axial length magnification on cpRNFL measured by various OCT devices has been previously described^(17,20)^. Our findings are in line with previous studies showing high myopic eyes have thicker temporal cpRNFL^(21)^, which has been attributed to RNFL redistribution as a result of the eye elongation that drags the retina toward the temporal horizon^(21,22)^.

Prior to correction of the magnification effect, there was a negative correlation between GCIPL and axial length, but following correction, the correlation became positive. Thus, applying the correction factor reversed our initial results, supporting the influence of ocular axial length on cpRNFL and GCIPL thickness when measured by Cirrus HD-OCT. The current diagnosis, evaluation, and follow-up of glaucoma patients require the evaluation of the thickness of cpRNFL and GCIPL, using OCT, which provides good sensitivity and specificity for the detection of glaucomatous damage^(3,4,23)^. Abnormal findings associated with a longer axial length were also observed in healthy eyes^(6,7,24)^. The normal database for cpRNFL and GCIPL measurements packaged within the Spectral-OCT software did not include individuals with moderate or high degrees of myopia^(14)^. As seen in our study, longer axial length led to an artificial thinning without the proper correction for magnification effect. As such, the color code classification, which is based on the comparison with the normative database, can lead to labeling non-glaucomatous myopic individuals as glaucoma suspects.

A decrease in cpRNFL^(11-13)^ and GCIPL thickness^(6,7,25)^ with increasing axial length has been reported in several studies. Retinal thinning in myopic eyes was speculated to result from mechanical stretching of the sclera along with axial elongation^(11,24)^. However, these previous investigations did not consider the effect of ocular magnification. Although our findings before the magnification effect correction are in accordance with these studies, we suggest a careful interpretation of the data, since our results support that the effect of retinal thinning might be in part an artefact due to ocular magnification factors and not because of a real anatomical change.

One of the proposed explanations is that the actual diameter of the OCT’s scan circle projected onto the retina is larger in eyes with longer axial length^(17,20,25)^. Due to the considerable impact of ocular magnification effect on the cpRNFL and macula measurements, routine correction for this factor has been suggested^(17,18,25)^. We used axial length to correct for ocular magnification as described by Bennett et al.^(16)^, wherein the Littmann’s formula, a reliable correction formula for fundus imaging^(16-18)^, was modified. This modified method is the most reliable of all methods and is convenient to use since it requires no data other than the eye’s axial length^(16)^.

To the best of our knowledge, there has been only one previous study describing the influence of axial length and magnification effect on GCIPL thickness measured by Cirrus-OCT^(26)^; however, our results are somewhat contradictory to their findings. Ueda et al. evaluated the effects of axial length on cpRNFL and GCIPL thickness using three spectral-domain OCT devices. Similar to our findings, when using Cirrus-OCT, the authors found a significant negative correlation between GCIPL thickness and axial length before magnification effect correction that turned into a significant positive correlation after the correction. Additionally, they found a positive correlation between temporal cpRNFL and axial length before and after correction for magnification, whereas we only found this positive correlation only after the correction. Also, a positive correlation between average cpRNFL and axial length after the correction was found, which we did not observe in our study. These discrete discrepancies may be attributed to differences in the study population; Ueda et al.^(26)^ assessed an older population with a higher average axial length compared to our population. Nevertheless, these findings highlight the importance of magnification effect correction to accurately measure cpRNFL to avoid a misleading diagnosis.

Nakanishi et al. investigated the effect of axial length-related ocular magnification on the thickness of the macular ganglion cell complex (mGCC) and showed that, in normal eyes, the inferior mGCC was negati vely correlated with the axial length after magnification correction. However, they used a different SD-OCT device and software (RS-3000 Nidek), and also assessed a population with higher axial length than our population^(25)^. Öner et al.^(20)^ described the thinning of cpRNFL in myopic individuals when compared to hyperopic and controls measured by Stratus-OCT (time-domain) and showed that this correlation disappeared when correction for ocular magnification effect was applied, which is in line with our findings^(20)^. Our finding of positive correlations between axial length and temporal RNFL and average GCIPL thickness, which was not seen in Öner’s study, may be due to better image acquisition speed, higher resolution and greater volume of data acquired with each scan using Cirrus-OCT (spectral-domain), when compared to the time-domain OCT^(27)^. Cirrus HD-OCT has a short scan time and can detect eye movement during imaging, which prevents misalignment of the scan circle that can affect the cpRNFL thickness assessment^(17,28)^. Another factor that can lead to misguided cpRNFL measurements is severe peripapillary atrophy or tilt^(29)^. However, the subjects enrolled in this study did not have peripapillary atrophy beyond the scanning circle, and only two eyes had temporally tilted discs (2,4%).

In our study, all images were acquired by a single operator, with high-quality and high-repetition scanning on the current generation OCT and optical coherence biometry instruments, leading to high internal validity. Additionally, previous studies demonstrated a progressive age-related decline of RNFL and GCIPL thickness detected with OCT imaging^(13,26)^, therefore we adjusted our analysis to account for this effect. The subjects enrolled were homogenous, healthy, of uniform age, and ethnicity (Caucasian) and had a reasonable range of axial length (21.39-26.72 mm), resulting in a refractive error range from -5,75 to +5,75 spherical equivalent diopters including myopic, emmetropic, and hyperopic individuals.

Limitations of the current study included restricted external validity due to its almost entirely Caucasian population, since differences may exist among ethnic groups. Another limitation is that only 10% of the enrolled subjects had spherical equivalents greater than four diopters, due to enrollment through volunteer enlistment. Therefore, our generalizability of the effect of long axial lengths on the thickness of cpRNFL and GCIPL was limited. Additionally, we did not evaluate the influence of different optic disc diameters on cpRNFL and GCIPL measurements. Seo et al., showed thicker superior and nasal cpRNFL, but not GCIPL, in individuals with larger optic disc, which was speculated to be due to overestimated scan circle^(21)^; however our current study was designed for a different purpose and is perhaps a topic for future studies.

In summary, our findings suggest that the reported effects of axial length on cpRNFL thickness cannot be interpreted correctly without considering magnification factors. It is plausible that individuals with longer axial length might present with thinning of cpRNFL and GCIPL due to anatomical changes related to ocular stretching. However, this may be overestimated if the effect of ocular magnification adjustment is not factored into the analysis. Therefore, we recommend careful interpretation of cpRNFL and GCA data particularly derived from moderate to highly myopic individuals when using OCT devices currently available. This approach can avoid misdiagnosing glaucomatous changes in myopic eyes. Additionally, we recommend that either ophthalmologists correct for ocular magnification effect or current Cirrus HD-OCT should be improved according to axial length. However, further studies are required to investigate the impact of ocular magnification correction on the diagnostic accuracy of Cirrus HD-OCT.
